# Elevated circulating CD14^++^CD16^+^ intermediate monocytes are independently associated with extracardiac complications after cardiac surgery

**DOI:** 10.1038/s41598-020-57700-9

**Published:** 2020-01-22

**Authors:** Jana C. Mossanen, Tobias U. Jansen, Jessica Pracht, Anke Liepelt, Lukas Buendgens, Christian Stoppe, Andreas Goetzenich, Tim-Philipp Simon, Rüdiger Autschbach, Gernot Marx, Frank Tacke

**Affiliations:** 1 Department of Medicine III, University Hospital Aachen, Aachen, Germany; 2 Department of Intensive and Intermediate Care, University Hospital Aachen, Aachen, Germany; 3 Department of Thoracic and Cardiovascular Surgery, University Hospital Aachen, Aachen, Germany; 4 Department of Hepatology & Gastroenterology, Charité University Medical Center, Berlin, Germany

**Keywords:** Cellular immunity, Cardiac device therapy

## Abstract

Elective cardiac surgery has low procedural complications. However, about 40% of patients develop extracardiac complications including delirium and acute kidney injury. We hypothesized that inflammatory processes and immune cell activation might be associated with these complications. We therefore prospectively included 104 patients undergoing cardiac surgery in our study. We assessed peripheral blood leukocyte populations by flow cytometry and circulating cytokines before operation, after surgery and at days one and four post-operatively. Patients undergoing cardiac surgery showed significantly elevated leukocytes and neutrophils after surgery. On the contrary, monocytes decreased after surgery and significantly increased at days 1 and 4, particularly classical (Mon1,CD14++CD16−) and intermediate (Mon2,CD14++CD16+) monocytes. While peripheral leukocyte subsets were unaltered in patients with infectious (n = 15) or cardiac complications (n = 31), post-operative leukocytes (p = 0.0016), neutrophils (p = 0.0061) and Mon2 (p = 0.0007) were clearly raised in patients developing extracardiac complications (n = 35). Using multiple logistic regression analyses, patient’s age, ICU days, number of blood transfusions and elevated post-surgery Mon2 independently predicted extracardiac complications. Our findings demonstrate that elevated Mon2 after cardiac surgery are associated with an increased risk for extracardiac complications. These findings might improve the risk estimation after cardiac operations and the role of Mon2 for inflammation in cardiac surgery.

## Introduction

Despite advances in surgical and anesthetic techniques as well as enhanced postoperative care, cardiac surgery is still associated with a high risk of postoperative complications^1^. Besides typical cardiac (e.g., arrhythmia, myocardial infarction) and infectious complications (e.g., wound infection, pneumonia and sepsis), extracardiac non-infectious complications like acute kidney injury (AKI, 30%) and delirium (up to 26–52%) are frequent in patients after cardiac surgery^2,3^. It has been speculated that a large part of these complications could be explained by excessive inflammation caused by extracorporeal cardio-pulmonary bypass (CPB), hypothermia, myocardial ischemia and reperfusion and tissue damage due to the surgical procedure^4,5^. The influence of distinct immune cells and their subpopulations on the occurrence of post-operative complications is still ambiguous and needs further investigations. Particularly monocytes seems to play in important role within this context^6,7^.

Circulating monocyte subsets represent a continuum of differentiation stages^8,9^. Using the surface markers CD14 (LPS receptor) and CD16 (FcγRIII), blood monocytes can be subdivided into three subpopulations, CD14++CD16− “classical” monocytes (Mon1), CD14++CD16+ “intermediate” (Mon2) and CD14–16+ “non-classical” (Mon3) monocytes^10,11^. Monocytes are a key player in cardiovascular disease and atherosclerosis, and elevated CD14−CD16+ non-classical monocytes have been correlated with endothelial dysfunction and vascular oxidative stress^12,13^. Several smaller studies (comprising 10–20 patients) have reported alterations in monocyte populations after cardiac surgery^6,14^, but the pathogenic involvement and the clinical relevance of these findings have remained elusive. In order to identify associations between monocyte subpopulations and complications after cardiac surgery, we conducted a prospective observational trial in 104 consecutive cardiac surgery patients, in which we obtained detailed circulating immune cell characteristics by multi-color flow cytometry before and sequentially after surgery. We thereby demonstrate that elevated post-operative CD14++CD16+ intermediate monocytes are associated with an increased risk for extracardiac complications, which may indicate a novel role of Mon2 in the context of inflammation related to cardiac surgery.

## Methods

### Study design and patient characteristics

A total of 107 patients (77 male, 30 female) were consecutively enrolled in this prospective, observational study after approval of the local institutional review board and after obtaining informed consent (Clinical trials gov. identification: NCT02488876). Ethical permission has been obtained from the Research Ethics Committee of RWTH-University Aachen, Germany (EK 151/09). This study has been performed in accordance with the ethical standards in the Declaration of Helsinki. The cohort is a sub-study from our previously published study aiming at identifying novel predictive biomarkers for acute renal failure in this setting^15^.

Patients were included over a pre-specified time period from June1, 2014, to November 31, 2015 on  two pre-defined days of the week. Exclusion criteria were pregnancy, emergency operations and patients aged less than 18 years. Out of these 107 patients, 3 patients received “off-pump” cardiac surgery; these three patients were excluded from the analysis due a potentially different activation of immune cells that had not been exposed to extracorporeal circulation^16,17^. Patient data, clinical information and blood samples were collected prospectively immediately before (pre) surgery, after (post) surgery as well as 1 day and 4 days after cardiac surgery. Serum samples and whole blood sufficient for multicolor fluorescence activated cell sorting (FACS) analysis were available before surgery in 104/104, directly after surgery in 96/104, at day 1 in 95/104 and at day 4 in 78/104 patients. The clinical course of patients was observed during the follow-up period by directly contacting the patients, their relatives or their primary care physician. Survival was assessed at 30 and 90 days.

### Management of anesthesia and surgical procedures

Anesthesia was performed as described before regarding to our institutional routine^18^. After surgery, all patients were transferred to the intensive care unit (ICU), and the following post-operative treatment was standardized according to our institutional guidelines. The surgical procedure with use of conventional cardiopulmonary bypass (CPB, ‘on-pump’) was performed in accordance to local clinical standards. The extracorporeal circulation was performed with a non-pulsatile pump flow of 2.2 l/min/m^2^, targeting a blood pressure between 50 and 70 mmHg.

### Postoperative complications

Postoperative complication within the first four postoperative days were subdivided in infectious complications (sepsis, wound infection, pneumonia), cardiac complications (right heart failure, arrhythmia, myocardial infarction) and extracardiac complications (delirium and acute kidney injury (AKI)). Arrhythmia was defined as a novel incidence of atrial fibrillation or flutter, ventricular tachycardia and fibrillation within the first four days after surgery. For extracardiac complications, delirium was defined via the Confusion Assessment Method for the ICU (CAM-ICU) score, acute kidney injury was based on creatinine increase (KDIGO criteria). Other extracardiac complications like ARDS (acute respiratory distress syndrome) and severe liver failure were also recorded, but were so infrequent (with only one case) that they were not further included in the analyses.

To understand the potential influence of different immune cell populations on the development of the postoperative complications, we compared immune cell populations and subpopulations of patients with and without the pre-specified complications. Additionally, we analyzed immune cells of 20 control patients without known cardiac, extracardiac or infectious diseases to assess potential changes in immune cell populations in patients requiring cardiac surgery.

### Analyses of serum cytokines and blood leukocytes

Blood samples for serum analyses were immediately placed on ice and stored at −80 °C after centrifugation. Cytokine concentrations of IL1ß, IL2, IL4, IL6, IL8, IL10, IL12(p70) and IL17 were measured using the Bio-Plex Assay (Bio-Rad Laboratories), following the manufacturer’s protocol. 20 ml whole blood for immune cell analyzes was subjected to red cell lysis by Pharmlyse (BD) and stained with fluorochrome-conjugated antibodies for FACS analysis^19^. Cytokines concentrations were measured, because they might allow conclusions about systemic inflammatory responses as well as the activation of different immune cell populations.

### Flow cytometry

After obtaining the blood samples, peripheral blood mononuclear cells (PBMCs) were isolated and immediately analyzed, as described before^20^.

PBMCs were stained with fluorescence-conjugated antibodies (CD14, CD56, CD45, CD3, CD4, CD19 by eBioscience; CD16, CD8 by BD Pharmingen) and incubated light protected for 30 min. After washing and centrifugation, cells were measured using a FACS Canto-II (BD, Heidelberg, Germany) and analyzed by using FlowJo Software (TreeStar, Inc., Ashland, USA). Cell populations were determined after exclusion of doublets and by forward and side scatter (FSC, SSC) and further by positivity for specific surface markers. For monocytes subpopulations, we selected the monocyte population in the forward and side scatter and excluded in the next step CD56+ cells, followed by subsequent analysis of CD14 and CD16. Subpopulations were divided into Mon1 (CD14++CD16−), Mon2 (CD14++ CD16+) and Mon3 (CD14−CD16+). Absolute cell numbers were calculated using the relative proportions of the subsets (from FACS) and the automated differential white blood cell counts.

### Statistical analysis

All statistical analyses were performed using GraphPad Prism 5.0 (Graphpad Software Inc., San Diego, CA, USA) and SPSS 23 (SPSS Inc., Chicago, IL, USA), as described before^15^. Patient data are displayed as median and range, providing the skewed distribution of most parameters. For testing differences between the control group and the patients, we used the χ^2^ test and Mann–Whitney U test. Differences in longitudinal samples were tested by Wilcoxon test, correlations were assessed by Spearman rank correlation analysis. Univariate logistic regression analyses were performed for all monocytes subsets and cell populations, interleukin levels and clinical data regarding to different outcome parameters (infectious, cardiac and noncardiac complications). Multivariate logistic regression analysis was performed with significant parameters from univariate logistic regression analysis followed by backward elimination. All patients measured at the specific time point were includedin uni- and multivariate analysis. Only Box plot graphics illustrate comparisons between subgroups. They display a statistical summary of the median, quartiles and range. Receiver operating characteristic curves were generated by plotting sensitivity against 1-specificity. All values have been included in statistical analyses.

### Ethics approval and consent to participate

Clinical trials gov. identification: NCT02488876. Ethics committee RWTH-University, Aachen, Germany: EK 151/09.

## Results

### Patient characteristics and incidence of postoperative complications

The baseline clinical characteristics of 104 patients participating in our study are displayed in Table 1. The 30-day mortality was 1% (1/104), the 90-day mortality was 3% (3/104). 35 of 104 patients (33.7%) developed extracardiac complications, primarily acute kidney injury and delirium, as determined within the first four postoperative days (Table 2). Patients developing extracardiac complications after cardiac surgery were older, had a higher prevalence of (pre-existing) chronic kidney disease (CKD) and more frequently used aldosterone antagonists (Table 1). Three patients with CKD had dialysis (2 with extracardiac complications, 1 without). As expected, Sepsis-related organ failure assessment score (SOFA; day1) and Simplified Acute Physiology Score (SAPS; day 1 and 4) scores as well as time on ICU (intensive care unit) were higher in patients with than without post-operative extracardiac complications. In these patients, we found elevated total leukocytes, neutrophils and monocytes compared to patients without extracardiac complications. Compared to extracardiac complications, we observed fewer events of infectious (n = 15, 14.4%) and cardiac complications (n = 31, 29.8%) during the observation period in the total patients cohort (Table 2).

**Table 1:** Patients’ characteristics.

Parameter	All patients	No extracardiac complication	Extracardiac complication	*p-*Value*
	*n* = 104	*n* = 69	*n* = 35	
**Demographics**				
Sex (male/female)	75/29	51/18	24/11	0.645
Age median (IQR) (years)	69 (61–76)	66 (60–75)	75 (66–79)	**0.003**
Body mass index (IQR) (kg/m²)	27 (25–30)	27 (25–30)	27 (25–29)	0.633
30 day mortality n (%)	1 (1)	0 (0)	1 (3)	0.160
90 day mortality n (%)	3 (3)	1 (1)	2 (6)	0.222
**Surgery and ICU observation**				
CABG n (%)	53 (51)	40 (58)	13 (37)	**0.045**
CABG + VR/R n (%)	23 (22)	11 (16)	12 (34)	**0.033**
VR/R n (%)	20 (19)	11 (16)	9 (26)	0.232
Other (Bentall, David a.o.) n (%)	8 (8)	7 (10)	1 (3)	0.188
Ischemia time (IQR) (min)	78 (60–109)	78 (60–105)	75 (63–110)	0.758
Time of CPB (IQR) (min)	125 (100–156)	125 (105–149)	130 (97–160)	0.604
Total time of surgery (IQR) (min)	250 (210–290)	250 (220–285)	245 (210–315)	0.786
**Postoperative period**				
ICU days median (IQR) (days)	3 (2–5)	2 (2–3)	5 (3–7)	**<0.001**
SAPS day 1 median (IQR)	29 (25–35)	27 (24–33)	33 (28–37)	**0.010**
SAPS day 4 median (IQR)	24 (19–31)	21 (17–23)	27 (21–32)	0.070
SOFA day 1 median (IQR)	5 (3–7)	5 (2–6)	6 (4–7)	**0.002**
SOFA day 4 median (IQR)	0 (0–3)	0 (0–1)	3 (0–4)	**<0.001**
Myocardial infarction n (%)	1 (1)	1 (1)	0 (0)	0.474
Arrhythmia n (%)	29 (28)	15 (22)	14 (40)	0.05
Right heart failure n (%)	2 (2)	0 (0)	2 (6)	**0.045**
**Comorbidities**				
Diabetes n (%)	36 (35)	23 (33)	13 (37)	0.701
Hypertension n (%)	78 (75)	52 (75)	26 (74)	0.905
Chronic kidney disease n (%)	10 (10)	3 (4)	7 (20)	**0.011**
Chronic lung disease n (%)	14 (14)	8 (12)	6 (17)	0.357
**Medication pre surgery**				
Diuretics use n (%)	51 (49)	30 (43)	21 (60)	0.113
β-blocker use n (%)	78 (75)	53 (77)	25 (71)	0.551
AT II receptor antagonist use n (%)	24 (23)	14 (20)	10 (29)	0.346
ACE inhibitor use n (%)	51 (49)	35 (51)	16 (46)	0.631
Statin use n (%)	67 (64)	45 (65)	22 (63)	0.813
Calcium channel blocker use n (%)	25 (24)	14 (20)	11 (31)	0.211
Aldosterone antagonist use n (%)	14 (13)	6 (9)	8 (23)	**0.047**
Aspirin use n (%)	82 (79)	53 (77)	29 (83)	0.478
**Laboratory parameters pre surgery**				
Creatinine (IQR) (mg/dl)	0.95 (0.74–1.07)	0.94 (0.74–1.06)	0.97 (0.76–1.12)	0.467
eGFR (ml/min)	75.1 (62.4–89.7)	77.4 (64.8–89.6)	72.7 (47.9–90.1)	0.284
**Laboratory parameters post surgery**				
WBC med. (IQR) (/µl)	9000 (7100–12250)	8800 (6300–11200)	11500 (8500–14500)	**0.002**
Neutrophils med. (IQR) (/µl)	7611(5831–10272)	7251(5337–9190)	9287 (6882–11955)	**0.006**
Monocytes (Mo) med. (IQR) (/µl)	489 (323–772)	441 (303–712.5)	629 (361–959)	**0.041**
Mon1 monocytes med. (IQR) (/µl)	425.5 (267–655)	377.3 (254–646)	527 (325–761)	0.069
Mon2 monocytes med. (IQR) (/µl)	7.2 (4.2–13.7)	5.5 (3.7–10.3)	13.7 (6.4–21.7)	**<0.001**
Mon3 monocytes med. (IQR) (/µl)	3.81 (2.33–6.69)	3.70 (2.36–5.99)	5.38 (2.30–9.98)	0.127
Lymphocytes med. (IQR) (/µl)	844 (582–1224)	824 (591–1154.3)	899 (540–1359)	0.734

*Statistical significance for differences between patients without and with extracardiac complications.

ACE: Angiotensin-converting-enzyme; AT: Angiotensin; ASS: acetylsalicylic acid; CABG: Coronary artery bypass graft; eGFR: estimated glomerual filtrationreate; VR/R valve reconstruction/ replacement; CPB: Cardiopulmonary bypass; ICU: Intensive care unit; IQR: Interquartile range; med.: median; Mo: Monocytes; Mon1, CD14++CD16− monocytes; Mon2, CD14++CD16+ monocytes; Mon3, CD14−CD16+ monocytes; OP: Operation; SAPS: Simplified acute physiology score; SOFA: Sequential organ failure assessment; WBC: White blood cell count.

**Table 2:** Post-operative complications.

Complications	n	%
**Infectious complications**	15	14.4
Wound infection	5	4.8
Sepsis	8	7.7
Pneumonia	12	11.5
**Cardiac complications**	31	29.8
Myocardial infarction	1	1.0
Arrhythmia	29	27.9
Right heart failure	2	1.9
**Extracardiac complications**	35	33.7
Acute kidney injury (AKI)	22	21.2
Postoperative delirium	22	21.2

### Alterations in circulating immune cell populations in patients undergoing cardiac surgery

**Figure 1:**
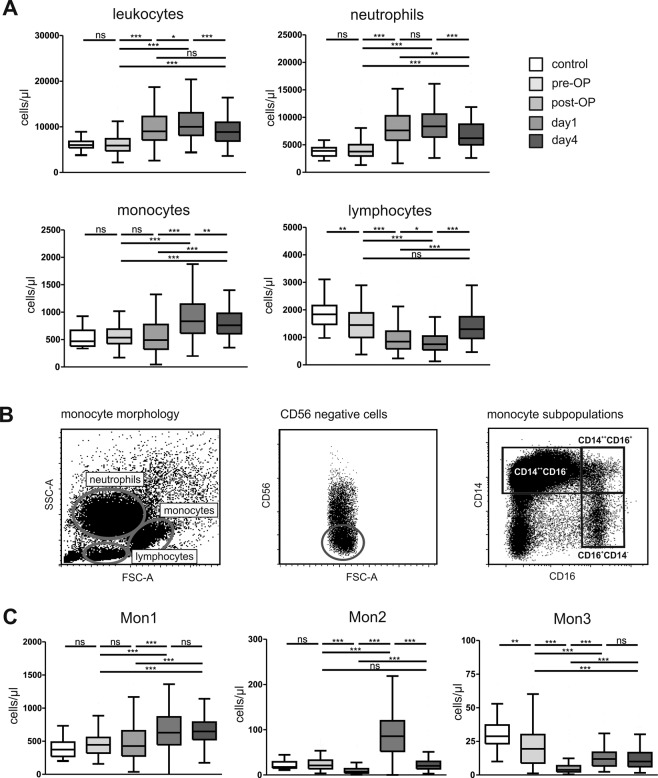
Characterization of immune cells in peripheral blood of patients undergoing cardiac surgery. (**A**) Immune cells (leukocytes, neutrophils, monocytes and lymphocytes) of patients undergoing cardiac surgery (n = 104) were analyzes before and immediately after cardiac surgery, at day 1 and 4 after cardiac surgery and compared to healthy controls (n = 23). (**B**) Representative gating of monocyte subpopulations into Mon1 (CD14++CD16− classical), Mon2 (CD14++CD16+ intermediate) and Mon3 (CD14−CD16+ non-classical) monocytes using flow cytometry. (**C**) Monocyte subpopulations in the peripheral blood of healthy controls and patients undergoing cardiac surgery at the different time-points (pre-operation, post-operation, day 1 and 4). *p < 0.05, ^**^p < 0.01, ^***^p < 0.001.

Immune cells gained from peripheral blood were analyzed before operation, immediately after cardiac surgery (at admission to the ICU) and at day one and four after cardiac surgery. Compared to healthy controls, patients undergoing cardiac surgery showed similar levels of leukocytes, neutrophils and monocytes before operation (at baseline), while lymphocytes were significantly reduced in patients undergoing cardiac surgery compared to healthy controls (Fig. 1A). Post-surgery levels of leukocytes and neutrophils increased, but showed a reduction between post-operative day 1 and 4. On the contrary, lymphocytes decreased within the first post-operative period and were restored to baseline levels at day 4 after surgery. Monocytes slightly decreased post-operatively and showed a significant increase at day 1 and day 4 compared to the pre-surgery value (p < 0.001) (Fig. 1A). We next analyzed monocyte subpopulations from peripheral blood (gated on FSC/SSC profile and negativity for CD56), based on CD14 and CD16 expression (Fig. 1B). This allowed us to differentiate between circulating Mon1 (CD14++CD16−), Mon2 (CD14++CD16+) and Mon3 (CD14−CD16+) monocyte subpopulations. Mon1 and Mon2 monocytes peaked at day 1 after surgery (Fig. 1C). While Mon1 remained high at day 4 after operation, Mon2 decreased between day 1 and day 4 (Fig. 1C). Mon3 were decreased at every time point after operation compared to the pre-operative value (Fig. 1C).

### No significant changes in circulating immune cell populations of patients developing infectious or cardiac complications

**Figure 2:**
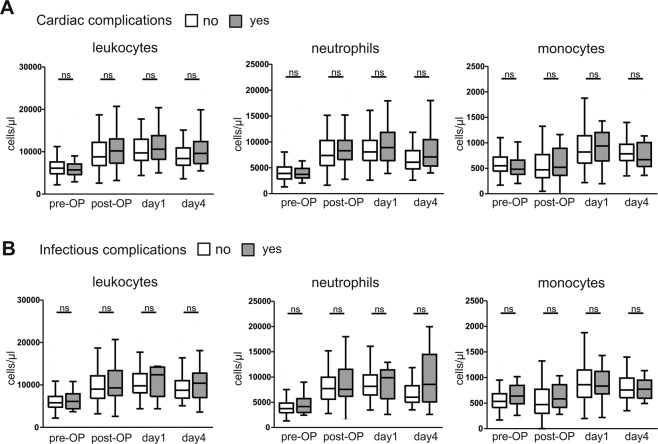
Characterization of immune cells in peripheral blood of patients developing cardiac or infectious complications after cardiac surgery. (**A**) Immune cell populations (leukocytes, neutrophils, monocytes) of patients undergoing cardiac surgery comparing patients developing cardiac complications (grey) to patients without cardiac complications (white), pre-operation, post-operation and at day 1 and 4 after surgery. (**B**) Immune cell populations of patients developing infectious complications (grey) compared to patients without infectious complications (white) during the first 4 days after surgery. *p < 0.05, ^**^p < 0.01, ^***^p < 0.001.

Comparing patients developing cardiac complications (n = 31, 29.8%) to patients without cardiac complications, immune cells did not differ within the two groups at the various time points (Fig. 2A, and data not shown). Moreover, patients with infectious complications (n = 15, 14.4%) after cardiac surgery showed no significant changes in the different immune cell populations in peripheral blood compared to patients without infectious complications (Fig. 2B).

### Significantly elevated leukocytes, neutrophils and Mon2 in patients developing extracardiac complications after surgery

**Figure 3:**
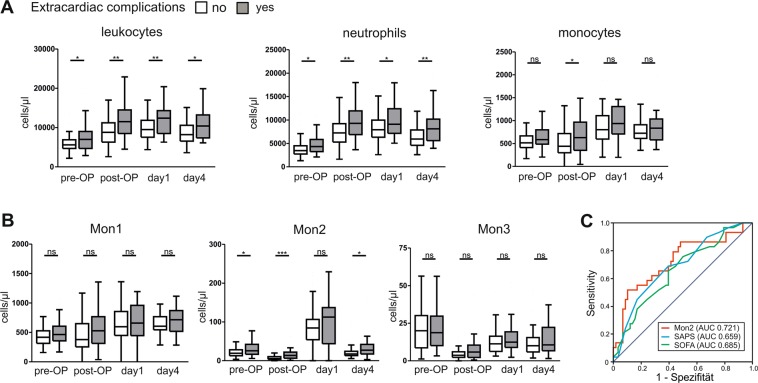
Characterization of immune cells and monocyte sub-populations of patients developing extracardiac complications. (**A**) Immune cells (leukocytes, neutrophils, monocytes) of patients undergoing cardiac surgery comparing patients developing extracardiac complications (grey) to patients without extracardiac complications (white), pre-operation, post-operation and at day 1 and 4 after operation. (**B**) Monocyte sub-populations of patients with (grey) or without (white) extracardiac complications at different time points before and after surgery. *p < 0.05, ^**^p < 0.01, ^***^p < 0.001. (**C**) Receiver operating characteristic (ROC) curve analyses for Mon2 (post-operation), SAPS and SOFA (both day 1) to predict extracardiac complications.

Comparing patients with and without extracardiac complications, leukocytes and neutrophils were significantly elevated at every time point in patients with complications, while monocytes were higher at the time point immediately after surgery (Fig. 3A). Among the different monocyte subpopulations, Mon2 were significantly higher in patients with extracardiac complications at every time-point, except for day 1 where the difference did not reach statistical significance (Fig. 3B). In contrast, Mon1 and Mon3 subpopulations did not differ significantly between the two groups at any time point (Fig. 3B).

### Correlations between monocyte subpopulations, inflammatory cytokines and clinical outcome parameters

In order to better understand the association between different monocyte subpopulations and extracardiac complications after surgery, we performed correlation analyses for all monocyte populations obtained at the four time-points with laboratory and clinical parameters (Table 3). Baseline levels of Mon2 and Mon3 monocytes correlated with hospitalization time, and baseline Mon1 as well as Mon2 correlated with interleukin 6 (IL-6) levels. Directly after surgery, Mon1 correlated with surgery time and time on extracorporeal circulation, suggesting that these procedures mobilize Mon1 monocytes. Mon3 after surgery inversely correlated with manifold inflammatory cytokines (IL-1β, IL-4, IL-6, IL-8, IL-12p70, IL-17), indicating activated (apoptotic) Mon3 as a potential preceding source of these cytokines^21^. Moreover, Mon1 were also correlated with surgery time and time of CPB (at the post-operative time point, while Mon2 were correlated to fluid balance and hospitalization time at day 4 after cardiac surgery. Thereby the postoperative significant decrease of Mon2 compared to the pre-operative value does not correlate to fluid balance, wherefore dilutions caused by the surgery procedure might not influence the findings at the post-operative time point.

**Table 3:** Correlation of different monocyte subpopulations at different time points with clinical parameters and cytokines.

Cell population	Correlation with	pre-OP		post-OP		day 1		day 4	
		r	p	r	p	r	p	r	p
Mon1 monocytes	Surgery time			0.264	0.010			0.249	0.038
	Time of CPB			0.280	0.006				
	IL-6	0.246	0.014						
	IL-8					−0.225	0.027		
	IL-17			−0.326	0.001				
Mon2 monocytes	Days of Hospitalization	0.258	0.009					0.310	0.008
	Time of CPB	−0.241	0.016						
	Fluid balance							0.395	0.001
	IL-4			0.270	0.008				
	IL-6	0.273	0.006						
	IL-17			−0.351	0.001				
Mon3 monocytes	Days of Hospitalization	0.280	0.005					−0.280	0.018
	Time of CPB	−0.234	0.020						
	Fluid balance					0.241	0.018		
	IL-1β			−0.258	0.012				
	IL-4	−0.238	0.017	−0.334	0.001				
	IL-6			−0.326	0.001	0.248	0.014		
	IL-8			−0.326	0.001	0.237	0.020		
	IL-12p70			−0.245	0.017				

CPB: Cardiopulmonary bypass, IL: Interleukin; Mon1, CD14++CD16− monocytes; Mon2, CD14++CD16+ monocytes; Mon3, CD14−CD16+ monocytes.

Days of Hospitalization: Overall time of stay in hospital; Fluid balance: post-OP – Fluid balance ad admission at the ICU (measured since arriving at the operation theater), day 1 and day 4: fluid balance within the last 24 hours.

To evaluate if elevated Mon2 after surgery predict extracardiac complications, we compared Mon2 post operation, SAPS and SOFA score at day 1 by receiver operating characteristic (ROC) curve analyses (Fig. 3C). Specifically elevated Mon2 were a predictor for extracardiac complications after cardiac surgery.

### Elevated post-operative Mon2 are independently associated with extracardiac complications in patients after cardiac surgery

Significantly elevated Mon2 after cardiac surgery in patients developing extracardiac complications and the correlation of this monocyte subpopulation at baseline to days of hospitalization and to IL6 led us to hypothesize that circulating monocyte populations might predict extracardiac complications after cardiac surgery. In order to analyze the predictive value of Mon2 for post-operative extracardiac complications, we conducted uni- and multivariate logistic regression analyses. By univariate analysis, several clinical characteristics and immune cell populations, particularly neutrophils and monocytes, at different time-points were associated with the occurrence of extracardiac complications (Table 4). By multivariate regression, only patient’s age, the administered number of red blood cell packages, ICU days and post-operative Mon2 remained independent predictors for extracardiac complications in patients undergoing cardiac surgery (Table 4). By multivariate regression analyses that included all monocytes subsets and all outcomes, only Mon2 remained an independent predictor for extracardiac complications pre surgery (p = 0.047) and post surgery (p = 0.025).

**Table 4:** Uni- and multivariate logistic regressions analyses for clinical and laboratory parameters to predict extracardiac complications after cardiac surgery.

	Parameter	Univariate OR (95%-CI)	p-value	Multivariate OR (95%-CI)	p-value
Basics	Age (years)	1.057 (1.012–1.105)	**0.013**	1.078 (1.015–1.144)	**0.04**
	Operation involving valve	2.334 (1.012–5.384)	**0.047**		n.s.
	CABG	0.428 (0.186–0.988)	**0.048**		n.s.
	Red blood cell packages (n)	1.318 (1.070–1.624)	**0.009**	1.338 (1.042–1.719)	**0.023**
	Chronic kidney disease (n)	5.500 (1.326–22.820)	**0.019**		n.s.
	ICU days (n)	1.934 (1.447–2.584)	**<0.001**	1.908 (1.374–2.651)	**<0.001**
Pre-Op	Leucocytes preoperative (1/µl)	1.000 (1.000–1.000)	**0.027**		n.s.
	Neutrophiles preoperative (1/µl)	1.000 (1.000–1.000)	**0.032**		n.s.
	Monocytes preoperative (1/µl)	1.002 (1.000–1.004)	**0.045**		n.s.
	Mon1 (1/µl)	1.002 (1.000–1.005)	**0.044**		n.s.
	Mon2 (1/µl)	1.029 (1.003–1.056)	**0.027**		n.s.
Post-OP	Leucocytes postoperative (1/µl)	1.000 (1.000–1.000)	**0.001**		n.s.
	Neutrophils postoperative (1/µl)	1.000 (1.000–1.000)	**0.003**		n.s.
	Monocytes postoperative (1/µl)	1.001 (1.000–1.003)	**0.039**		n.s.
	Mon2 (1/µl)	1.092 (1.032–1.156)	**0.002**	1.106 (1.036–1.181)	**0.003**
	Mon3 (1/µl)	1.076 (1.007–1.150)	**0.03**		n.s.
	Lactate postoperative (mg/dl)	1.448 (1.074–1.952)	**0.015**		n.s.
1.POD	Leucocytes on first POD (1/µl)	1.000 (1.000–1.000)	**0.001**		n.s.
	Neutrophils on first POD (1/µl)	1.000 (1.000–1.000)	**0.008**		n.s.
	Lymphocytes on first POD (1/µl)	1.001 (1.000–1.002)	**0.031**		n.s.

CABG, Coronary artery bypass graft; CI, confidence interval; ICU, intensive care unit; OR, odds ratio; POD, post-operative day; Mon1, CD14++CD16− monocytes; Mon2, CD14++CD16+ monocytes; Mon3, CD14−CD16+ monocytes.

## Discussion

In this study, we comprehensively evaluated the kinetics of circulating immune cell populations and inflammatory cytokines before and during the ICU course in a large, prospectively enrolled cohort of patients undergoing elective cardiac surgery in order to identify dysregulated immunological and inflammatory pathways related to the development of clinical complications^4^. While we did not identify such associations with infectious or cardiac complications, manifold inflammatory and immune pathways appeared dysregulated before or immediately after surgery between patients with or without extracardiac complications. Among these complex alterations, post-operatively measured circulating Mon2 independently predicted extracardiac complications within the first four days after surgery.

Monocytes are involved in many pathogenic processes, ranging from the acute response to injury and infections to modulation of chronic inflammation (as, for instance, seen in atherosclerosis) to wound healing^22,23^. The latter aspect appears particularly relevant for the healing phase after myocardial injury in the heart^24,25^. Novel technologies like single-cell RNA sequencing provided further in-depth insights into the heterogeneity of monocytes in human blood^26^. Three major human monocyte subpopulations have been described^27^, of which the Mon1 (classical monocytes) are relatively immature and preferentially recruited to injured tissue, while Mon2 (intermediate) and Mon3 (non-classical monocytes) have a higher capacity to secrete cytokines upon activation. Interestingly, in several specific patient populations with atrial fibrillation, heart failure and chronic kidney disease, Mon2 were an independent predictor for cardiovascular events^28–30^. Elevated Mon2 have been reported before in patients undergoing cardiac surgery^14^. Another study emphasized the relevance of CD16 expression of circulating monocytes by measuring the mean fluorescence index (MFI) of CD16 in patients undergoing cardiac surgery pre-operation and at day 5 and 90. In agreement with our data, elevated MFI of CD16 was observed at day 5 after operation^6^. This small study (n = 17 patients), however, did not identify an association between CD16 and outcome^6^. In a larger study, Mon3 have been associated with advanced vascular dysfunction in patients undergoing cardiopulmonary bypass^13^.

Regarding extracardiac complications, inflammatory monocytes have indeed been implicated in the pathogenesis of delirium and acute kidney injury. Data from mice with sepsis-associated encephalopathy, an established model of delirium, demonstrated reduced neuroinflammation accompanied with reduced signs of cognitive impairment by prevention of CCR2 + monocyte recruitment^31^. The blockade of CCR2 and thereby the reduced recruitment of inflammatory monocytes was also found to be protective in models of renal inflammation in mice^32,33^. Mon2 (intermediate) monocytes were described to be a predictor for cardiovascular disease events in CKD patients with or without hemodialysis^28,34^. Regarding to non-dialysis patients there were no elevated Mon2 compared to patients with preserved renal function^28^. Patients with dialysis had higher numbers of Mon 2 as part of inflammatory monocytes compared to patients without renal dysfunction^34^.

In our analyses, Mon1 and Mon2 correlated with IL6 levels before surgery. Other studies could show that patients with delirium hat significant higher blood cytokine levels (including IL6) comparing to those without delirium^35^. On the other hand, IL6 mediated neutrophil activation is one of the central mechanisms in AKI^36^. Mon2 produce IL6 under stimulation, which may possibly explain the association of elevated Mon2 with extracardiac complications such as delirium and AKI after cardiac surgery. In this regard, it is unexpected that Mon2 were not associated with infectious complications. This could be potentially related to the low number of infections in our cohort (only 15 patients) as well as to the sampling time (up to 4 days after surgery might be still dominated by the procedure-related inflammatory reaction).

Univariate logistic regression analyses showed that elevated Mon 2 before and after surgery were predictive for extracardiac complications within the first four days after cardiac surgery. In would be of high interest, if measurement of Mon2 in the context of cardiac surgery might be an opportunity to prevent delirium and AKI by modulating immune cells and or eliminating certain cytokines. Until now it is ambiguous at which time point (before, while or after surgery) Mon2 levels might have the best predictive value.

Altogether, our data as well as findings from other studies support the hypothesis that circulating monocyte populations are indicative of activated immunological and inflammatory pathways, thereby supporting their potential as a biomarker as well as a potential therapeutic target. Many efforts currently focus on reversing “immune cell dysfunction” in patients with critical illness and/or sepsis; these approaches include the administration of immune modulators (e.g., G-/GM-CSF), removal of inflammatory cytokines (e.g., by synthetic haemabsorption columns) or cell-based therapies (e.g., mesenchymal stem cells or myeloid cells)^37^. Our study indicates that even patients undergoing elective cardiac surgery might benefit from implementing immune-related markers in risk stratification.

## Limitations

Our study was a single center study with a limited number of patients and was designed to assess 90-day mortality, but no longer follow-up periods. Due to the very low 90 day-mortality rate (3%), the sample size did not allow to assess the prognostic impact of leukocyte populations on survival. It can be anticipated that larger prospective multicenter studies may allow to better define the predictive value of elevated Mon2 monocytes after surgery and subdivide the extracardiac complications in distinct subgroups of AKI and delirium. To understand the effects of Mon2 monocytes as a pathophysiological component for the development of extracardiac complications such as AKI and delirium, the interaction between these cells and the surfaces of extracorporal circulation has to be analyzed in detail.

## Conclusion

Based on a prospectively enrolled cohort of 104 patients with sequential, detailed immune phenotyping analyses, our study identifies post-operatively elevated Mon2 (CD14++CD16+, intermediate) monocytes as a predictive marker for extracardiac complications in patients undergoing elective cardiac surgery. Our data support the hypothesis that immunological alterations during and after surgery are related to the subsequent clinical course of surgical patients and that analyzing monocyte subpopulations might be a promising biomarker for the development of post-operative complications. Despite their different pathophysiological background, we focused on acute kidney injury and delirium, because these are the most common non-infectious extracardiac complications after cardiac surgery. Larger multi-center studies are warranted to develop new strategies involving this marker in individual risk prediction for specific (possibly even more rare) extracardiac complications after elective heart surgery and for exploring novel therapeutic targets.
